# Rhinolithiasis as cause of oronasal fistula

**DOI:** 10.1016/S1808-8694(15)31294-5

**Published:** 2015-10-20

**Authors:** Gabriel Cesar Dib, Rodrigo P. Tangerina, Carlos E.C. Abreu, Rodrigo de Paula Santos, Luiz Carlos Gregório

**Affiliations:** 1 Specialization in Otorhinolaryngology under course, Federal University of Sao Paulo – Escola Paulista de Medicina; 2 Resident Physician in Otorhinolaryngology, Federal University of Sao Paulo – Escola Paulista de Medicina; 3 Master studies in Otorhinolaryngology under course, Federal University of Sao Paulo – Escola Paulista de Medicina; 4 Master; Ph.D. studies in Otorhinolaryngology under course, Federal University of Sao Paulo – Escola Paulista de Medicina; 5 Head of the Discipline of Otorhinolaryngology, Sector of Rhinology, Federal University of Sao Paulo – Escola Paulista de Medicina

**Keywords:** rhinolithiasis, rhinolith, oronasal fistula, nasal obstruction

## Abstract

Rhinolithiasis is a disease caused by deposition of organic and inorganic compounds in the nasal cavity, leading to unilateral nasal obstruction, fetid rhinorrhea, epistaxis, and it may cause complications. The authors present a case of rhinolithiasis with oronasal fistula and literature review.

## INTRODUCTION

Rhinolithiasis is an uncommon disease that may present asymptomatically, characterized by presence of mineralized tumor in the nasal cavity, which may be large and deviate neighboring structures^1^.

The presence of deviation and nasal septum perforation, destruction of nasal cavity lateral wall, involvement of maxillary sinus and production of oroantral or oronasal fistula are rare complications.

We report one case of rhinolithiasis with presence of oronasal fistula and present literature review on the condition.

## CASE REPORT

Female 43-year-old patient, Caucasian, single, housewife, born in Jacaúna-CE, living in Sao Paulo-SP, complained of left nasal obstruction for 11 years. She was seen in the outpatient clinic of Otorhinolaryngology, Hospital Sao Paulo, Federal University of Sao Paulo – Escola Paulista de Medicina.

She reported progressive nasal obstruction, only on the left nasal fossa, intermittent, that progressed to continuous obstruction, with anterior and posterior purulent discharge and cacosmia.

Six months before she had had perforation of hard palate, with drainage of nasal secretion into the oral cavity and regurgitation of liquids into the left nasal cavity. She did not report pain, nasal bleeding, headache, fever, loss of weight or allergic symptoms.

She reported that at the age of 2 years she introduced a bean seed into the left nostril, which was “removed the next day”, and she had remained without complaints up to the current presentation.

Rhinoscopy showed presence of purulent secretion and irregular surface tumor, which was gray and recovered by granulation tissue, stone-hard upon touch with scalpel, immovable, obstructing the left nasal fossa and affecting the floor, nasal septum, inferior and middle conchae, with nasal septum deviation to the right. The examination revealed extremely fetid odor from the nose.

**Figure 1:**
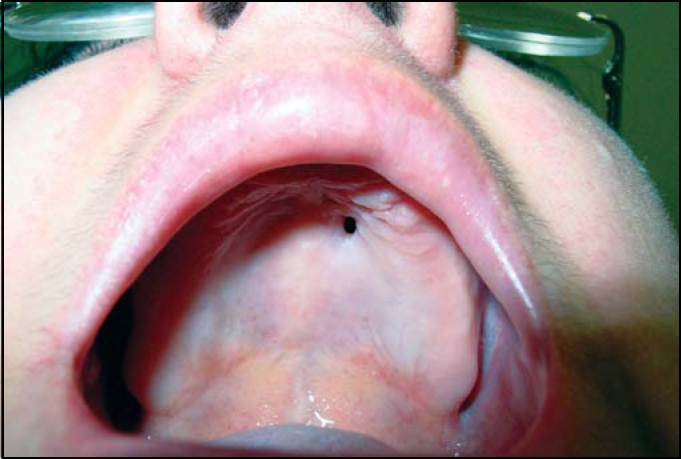
Oronasal fistula in the anterior region of hard palate on the left.

Oroscopy presented perforation in the left anterior region of hard palate, measuring 3 × 2 mm in diameter, with irregular margins, and drainage of purulent secretion into the oral cavity (Figure 1).

**Figure 2:**
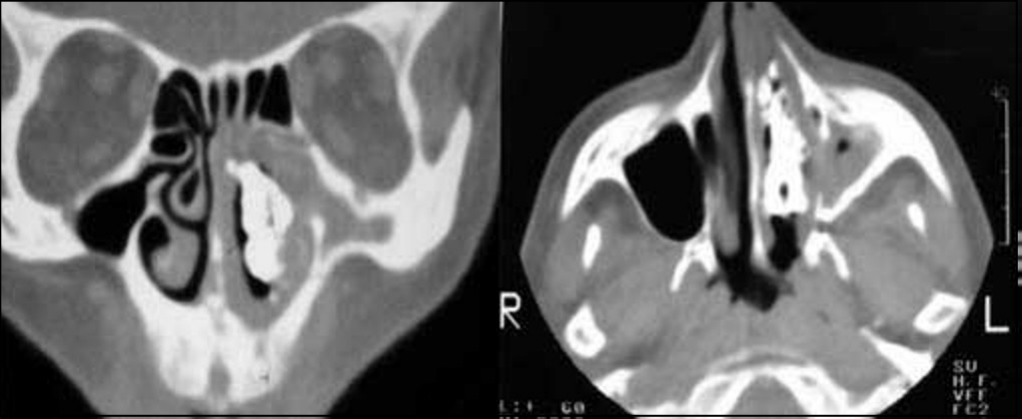
Coronal and axial CT scan sections showing large rhinolith in the left nasal fossa.

Paranasal sinuses CT scan revealed bone density tumor occupying the left nasal fossa (Figure 2).

It was not possible to perform nasofibroscopy because the tumor did not allow the passage of the instrument through the left nasal fossa.

**Figure 3:**
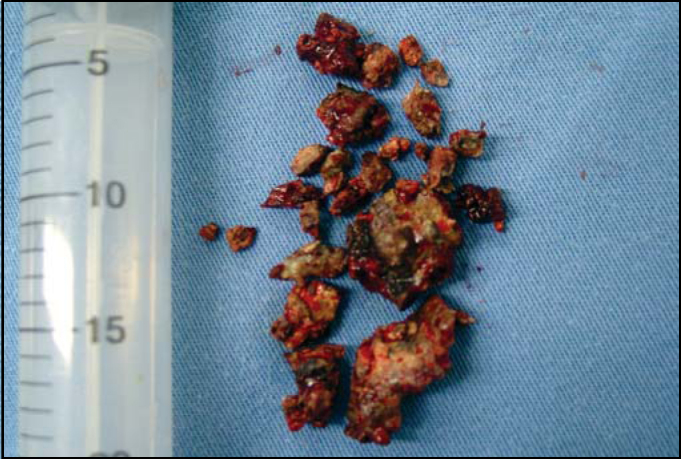
Surgical piece after fragmentation of rhinolith for its removal.

Based on the diagnostic hypothesis of rhinolithiasis, the patient was submitted to nasal endoscopic surgery and we removed a rhinolith measuring 4.5 × 2.5 × 1.5 cm, sent to clinical pathology analysis that evidenced chronic inflammatory process with granulation tissue and presence of filament bacteria suggestive of *Actinomices sp*. It was necessary to fragment the rhinolith so that it could be removed, owing to its extremely irregular shape and extension (Figure 3).

We decided not to close the oronasal fistula in the same surgical act owing to presence of marked local inflammatory process.

## DISCUSSION

Rhinolithiasis was first described by Bartholin in 1654. It is an uncommon affection that is many things left undetected by patients.

Etiology is not always detected, and it may be exogenous (such as grains, small stone fragments, plastic parts, seeds, insects, glass, wood and others), or endogenous, resulting from dry secretion, clots, cell lysis products, mucosa necrosis and tooth fragments, which operate as foreign body^2^, ^4^.

Foreign bodies normally access the site anteriorly, but they may occasionally reach into the nasal cavity through the choana owing to cough or vomiting^5^.

Foreign bodies are normally introduced during childhood, occupying the nasal floor in most situations^6^. Its presence causes local inflammatory reaction, leading to deposits of carbonate and calcium phosphate, magnesium, iron and aluminum, in addition to organic substances such as glutamic acid and glycin, leading to slow and progressive increase in size^4^, ^7^.

Symptoms are normally progressive unilateral nasal obstruction, rhinorrhea (usually purulent and fetid), cacosmia and epistaxis. Other less common symptoms are headache, facial pain and epiphora^8^, ^9^.

There may be complications such as nasal septum perforation or deviation, oroantral and oronasal fistula, chronic sinusitis and destruction of lateral nasal wall.

The physical examination showed gray and dark mass, with stone-hard consistency and irregular surface.

Diagnosis is normally based on symptomatology, history of foreign body introduction into the nose, physical examination and complementary tests. Simple x-ray and paranasal sinuses CT scan support the diagnosis through the presence of calcified tumor in the nasal fossa, in addition to supporting the planning of surgical approach^10^.

Diagnosis may be made through routine examination or revealed by imaging exam conducted by other reasons, such as for example a dental treatment^9^.

Differential diagnosis should take into account benign tumors (osteomas), bone sequestration and malignant tumors (chondrosarcoma, osteosarcoma, among others)^2^, ^11^.

Treatment consists of removal of rhinolith and the surgical approach chosen depends on location and size of the rhinolith and presence or not of complications, but most of them may be removed endonasally. External approaches may be necessary in cases of giant rhinoliths, and endoscopes are extremely helpful in both approaches^9^.

Treatment of complications can be performed in the same or in another surgical act^8^.

In the case of oronasal fistulas, there is a tendency in the literature to leave the correction to second intervention, which should be performed by rotation of palate and nasal flap, promoting two-layer closing^2^, ^11^.

## CLOSING REMARKS

Rhinolithiasis is an uncommon disease that may be left undiagnosed for many years and present complications. The diagnosis is normally made by clinical history and physical examination, and it should be considered in cases of unilateral nasal obstruction. Treatment consists of removing the rhinolith and correcting occasional complications.
